# Frequency, recurrence and factors associated with keratocysts diagnosed in the oral and maxillofacial pathology laboratory of the Universidad Peruana Cayetano Heredia Teaching Dental Center, Lima-Peru, 2000-2019

**DOI:** 10.21142/2523-2754-1404-2026-319

**Published:** 2026-10-01

**Authors:** Alex Sandra Rios Mendoza, Sujey Banda Mendoza, Carlos Espinoza Montes

**Affiliations:** 1 School of Stomatology, Cayetano Heredia Peruvian University. Lima, Peru. alex.rios@upch.pe sujey.banda@upch.pe carlos.espinoza@upch.pe School of Stomatology Cayetano Heredia Peruvian University Lima Peru alex.rios@upch.pe sujey.banda@upch.pe carlos.espinoza@upch.pe

**Keywords:** keratocysts, recurrence time, Gorlin-Goltz syndrome, jaws, queratoquistes, tiempo de recurrencia, síndrome de Gorlin-Goltz, maxilares

## Abstract

The keratocyst is a cystic lesion that originates from the remnants of the dental lamina. Its incidence is most common between the second and third decades of life, with a high prevalence in the lower jaw, particularly in the region of the last molars.

**Objective:**

To determine the frequency, recurrence of keratocysts, and associated factors in the oral and maxillofacial pathology laboratory of the dental teaching center at Universidad Peruana Cayetano Heredia (UPCH), Lima, Peru, during the period 2000-2019. Materials and Methods: This was a cross-sectional, analytical, descriptive, and retrospective study based on 15,926 histopathological records from the oral and maxillofacial pathology laboratory at UPCH (2000-2019). The sample applied inclusion and exclusion criteria.

**Results:**

A total of 321 cases of odontogenic keratocysts were analyzed, of which 75.08% were located in the lower jaw and 24.92% in the upper jaw. Within the lower jaw, the mandibular body was the most affected area (21.18%). Regarding recurrence, 11.53% of cases showed recurrence, most frequently within the first year (5.30%). The mandibular body was again the most affected site in recurrent cases (4.37%). In terms of sex distribution, there was a slight predominance in females (55.76%). The highest incidence was observed in patients aged 11 to 20 years (25.23%).

**Conclusion:**

Keratocysts predominantly affect the lower jaw, with the mandibular body being the most commonly involved area, particularly in young patients between 11 and 20 years old. Although recurrence is low, it primarily occurs within the first year after treatment. No significant differences were found in case distribution or recurrence based on sex or age group, suggesting that other factors may influence their development.

## RESUMEN

El queratoquiste es una lesión quística que se origina en los remanentes de la lámina dental. Su incidencia es más común entre la segunda y la tercera década de la vida, con una alta prevalencia en el maxilar inferior, particularmente en la región de los últimos molares.

**Objetivo:**

Determinar la frecuencia, recurrencia y factores asociados de los queratoquistes en el laboratorio de patología oral y maxilofacial del Centro Docente de Odontología de la Universidad Peruana Cayetano Heredia (UPCH), Lima, Perú, durante el período 2000-2019**.**

**Materiales y métodos:**

Estudio transversal, analítico, descriptivo y retrospectivo basado en 15,926 registros histopatológicos del laboratorio de patología oral y maxilofacial de la UPCH (2000-2019). La muestra aplicó criterios de inclusión y exclusión.

**Resultados:**

Se analizaron 321 casos de queratoquistes odontogénicos, de los cuales el 75,08% se localizaron en el maxilar inferior y el 24,92%, en el maxilar superior. Dentro del maxilar inferior, el cuerpo mandibular fue la zona más afectada (21,18%). En cuanto a la recurrencia, el 11,53% de los casos presentaron recurrencia, con mayor frecuencia dentro del primer año (5,30%). El cuerpo mandibular fue nuevamente el sitio más afectado en los casos recurrentes (4,37%). En cuanto a la distribución por sexo, hubo un ligero predominio en el sexo femenino (55,76%). La mayor incidencia se observó en pacientes de 11 a 20 años (25,23%).

**Conclusión:**

Los queratoquistes afectan predominantemente al maxilar inferior, siendo el cuerpo mandibular la zona más comúnmente involucrada, sobre todo en pacientes jóvenes entre 11 y 20 años. Aunque la recurrencia es baja, se presenta principalmente dentro del primer año después del tratamiento. No se encontraron diferencias significativas en la distribución de casos ni en la recurrencia según el sexo o el grupo de edad, lo cual sugiere que otros factores podrían influir en su desarrollo.

## INTRODUCTION

The odontogenic keratocyst (OKC) is a lesion of odontogenic origin characterized by its aggressive clinical behavior, high tendency for recurrence, and distinctive histological features. It was first described in 1956 as a cyst lined by keratinized stratified squamous epithelium ^7^. Decades later, in 1992, the World Health Organization (WHO) adopted the term “odontogenic keratocyst” as a synonym for “primordial cyst,” emphasizing its origin and particular histological characteristics ^1^.

In 2005, due to its clinical behavior, the identification of mutations in the PTCH1 tumor suppressor gene, the presence of satellite cysts, and its association with Gorlin-Goltz syndrome, the WHO proposed reclassifying it as a “benign keratocystic odontogenic tumor” (KCOT) ^2^^,^^16^. However, the 2017 revision returned it to the cyst category because there was insufficient evidence supporting its neoplastic nature. This decision was reaffirmed in the fifth edition of the classification published in 2022 ^3^.

The OKC accounts for approximately 10% of all odontogenic cysts ^3^^,^^4^. It generally arises from remnants of the dental lamina, although in a smaller proportion it may originate from basal epithelial cells or the reduced epithelium of the enamel organ ^1^. It is more frequently located in the mandible, particularly in the body and posterior region, and is mainly observed in young males between the second and third decades of life, with a second peak of occurrence in the fifth decade ^4^^,^^5^.

The keratocyst is often asymptomatic and is detected incidentally on radiographic examinations. However, in more advanced cases, it may cause bone expansion, facial asymmetry, pain, secondary infection, fistula formation, and even pathological fractures ^6^. Radiographically, it typically appears as a well-defined radiolucent image, generally unilocular, although larger lesions may be multilocular ^6^. Additionally, more than 40% of cases are associated with unerupted tooth crowns ^6^. Histologically, it presents a thin epithelial lining (6-8 cell layers), with palisaded basal cells, reversed nuclear polarization, and parakeratin within the cystic lumen ^19^.

Management of OKCs continues to be a subject of debate due to their high recurrence rate ^9^. Therapeutic alternatives range from conservative approaches, such as marsupialization and decompression-which help reduce lesion size and facilitate subsequent enucleation-to more radical procedures, such as ostectomy with fixative solutions (e.g., Carnoy’s solution) or cryotherapy ^9^^,^^10^. Despite these options, recurrence may range from 13.3% to 56%, generally due to the presence of satellite cysts, residual microcysts, or epithelial remnants ^15^^,^^17^^,^^18^.

Given its complex biology and clinical implications, the odontogenic keratocyst remains an important topic in both dental practice and scientific research. Understanding its prevalence, clinical and histological characteristics, and the factors involved in its development is essential for optimizing diagnosis, treatment, and long-term patient management.

## MATERIALS AND METHODS

A cross-sectional, analytical, descriptive, and retrospective study was conducted using non-probabilistic sampling and applying defined inclusion and exclusion criteria. The population consisted of 15,926 anatomopathological records registered in the Oral and Maxillofacial Pathology Laboratory of the Dental Teaching Center at Universidad Peruana Cayetano Heredia between the years 2000 and 2019. The final sample included 321 cases with a histopathological diagnosis of odontogenic keratocyst.

Cases with a definitive diagnosis between 2000 and 2019 that contained complete data (sex, age, and general location), as well as those without specific location, were included, since this variable was relevant for overall analysis. Cases associated with syndromes (e.g., Gorlin-Götz) and duplicate records were excluded, retaining only post-marsupialization information. Clinical and demographic variables were analyzed, including prevalence, general and specific anatomical location, recurrence, recurrence time and site, age group, mean age, and sex.

Ethical approval was obtained from CIEI-UPCH, as well as authorization from the corresponding Academic Department. The digital database and non-digitized physical records were reviewed, filtering diagnoses through assigned codes and extracting variables of interest. Data cleaning was performed using Microsoft Excel, and statistical analysis was conducted with Stata 17.0, applying descriptive analysis with absolute and relative frequencies for qualitative variables, measures of central tendency and dispersion for quantitative variables, and the Chi-square test with a 95% confidence level and p < 0.05.

The study was registered in SIDISI under code 213997 and approved by the Institutional Research Ethics Committee (CIEI) under approval code CIEI-312-27-24. Anonymity was ensured through correlational coding, without the use of personal identifiers.

## RESULTS

In this study, a total of 15,926 anatomopathological examinations from 2000 to 2019 were evaluated; however, only 321 cases (2.016%) corresponded to odontogenic keratocysts. These were classified according to anatomical location, recurrence, recurrence timing and site, age group, mean age, and sex of the patients.

The analysis of the general location of the lesions shows that 241 cases (75.08%) occurred in the lower jaw, while 80 cases (24.92%) occurred in the upper jaw. Regarding the specific anatomical location, the mandibular body was the most frequent site with 68 cases (21.18%). This was followed by the mandibular molar region with 35 cases (10.91%); the ramus with 22 cases (6.85%); the symphysis with 21 cases (6.54%); other areas of the maxilla with 16 cases (4.98%); incisors and canines with 15 cases (4.67%); other mandibular areas with 10 cases (3.13%); maxillary molars with 9 cases (2.80%); the angle with 6 cases (1.87%); parasymphysis with 5 cases (1.56%); and finally, the premolar region, which presented 0 cases (Table 1). Sex distribution showed a slight predominance in females, with 179 cases (55.76%) compared to 142 cases in males (44.24%). Regarding age groups, the data revealed that the 11-20-year group was the most affected, presenting 81 cases (25.23%); followed by 21-30 years with 55 cases (17.13%); 31-40 years with 53 cases (16.51%); 41-50 years with 43 cases (13.40%); 51-60 years with 31 cases (9.66%); ≥71 years with 26 cases (8.10%); 61-70 years with 25 cases (7.79%); and finally, the 1-10-year group with 7 cases (2.18%) (Table 1).

**Table 1:** Frequency and recurrence of keratocysts in the maxillofacial pathology laboratory of the UPCH teaching dental center, Lima-Peru during the period from 2000 to 2019

	Variables	n	%
General location			
	Maxilla	80	24,92
	Mandible	241	75,08
Specific location			
	No specific location	114	35,51
	Incisors and canines	15	4,67
	Premolars	0	0,00
	Molars (upper)	9	2,80
	Other areas of the maxilla	16	4,98
	Symphysis	21	6,54
	Parasymphysis	5	1,56
	Body	68	21,18
	Angle	6	1,87
	Branch	22	6,85
	Molars (lower)	35	10,91
	Other areas of the mandible	10	3,13
Recurrence			
	No	284	88,47
	Yes	37	11,53
Recurrence time			
	Not present	284	88,47
	Less than a year	0	0,00
	1 year	17	5,30
	2 years	4	1,25
	3 years	5	1,55
	4 years	4	1,25
	≥ 5 years	7	2,18
Lugar de recurrencia			
	No specific location	284	88,47
	Incisors and canines	2	0,62
	Premolars	3	0,93
	Molars (upper)	2	0,62
	Other areas of the maxilla	1	0,31
	Symphysis	2	0,62
	Parasymphysis	0	0,00
	Body	14	4,37
	Angle	1	0,31
	Branch	5	1,57
	Molars (lower)	7	2,18
	Other areas of the mandible	0	0,00
Sex			
	Female	179	55,76
	Male	142	44,24
Age group			
	01 to 10 years	7	2,18
	11 to 20 years	81	25,23
	21 to 30 years	55	17,13
	31 to 40 years	53	16,51
	41 to 50 years	43	13,40
	51 to 60 years	31	9,66
	61 to 70 years	25	7,79
	≥ To 71 years	26	8,10
Age			
X (DE)	19,15 (2.12)		
Total		321	100,00

n: absolute frequency. %: relative frequency. X: average. DE: standard deviation.

The distribution of odontogenic keratocysts is analyzed according to their general and specific location, considering variables such as sex and age group. Regarding general location, most cases were found in the lower jaw, with 241 cases (75.08%), while 80 cases (24.92%) occurred in the upper jaw. When analyzing the distribution of general location by sex, women presented 41 cases in the upper jaw (22.91%) and 138 cases in the lower jaw (77.09%). In men, 39 cases were recorded in the upper jaw (27.46%) and 103 in the lower jaw (72.54%). The difference between sexes was not statistically significant (p = 0.348). By age group, keratocysts were predominantly located in the lower jaw across all age ranges. The most frequent cases occurred in the 11-20-year group, with 61 cases in the lower jaw (75.31%) and 20 cases in the upper jaw (24.69%). The least frequent group was 1-10 years, with only 7 total cases: 6 in the lower jaw (85.71%) and 1 in the upper jaw (14.29%). Statistical analysis for age groups in relation to general location yielded a p-value of 0.527, indicating no statistically significant differences in the distribution of keratocysts between the upper and lower jaws based on age group. Regarding specific location by sex, the most frequent site in women was the mandibular body, with 42 cases (23.46%), and in men it was also the mandibular body (18.31%). The least frequent location was the premolar region, with 0 cases in both sexes. The analysis yielded a p-value of 0.069, indicating no statistically significant difference between sexes in the distribution of specific locations. Regarding specific location by age group within the jaws, the most affected group was 31-40 years in the mandibular body, with 16 cases (30.19%), followed by the 11-20-year group with 15 cases (18.52%). The least affected group was 1-10 years; however, when cases did occur, they were concentrated in the mandibular body (28.57%) and the molar region (14.29%). The analysis yielded a p-value of 0.477, indicating no statistically significant difference among age groups regarding specific anatomical location (Table 2).

**Table 2:** Location of keratocysts in the maxillofacial pathology laboratory of the UPCH teaching dental center, Lima-Peru during the period from 2000 to 2019

Variables		GENERAL LOCATION				SPECIFIC LOCATION					
		Maxilla		Mandible		P	No specific location		Incisors and canines		Premolars		Molars (upper)		Other areas of the maxilla		Symphysis		Parasymphysis		Body		Angle		Branch		Molars (lower)		Other areas of the mandible		p	
		n	%	n	%		n	%	n	%	n	%	n	%	n	%	n	%	n	%	n	%	n	%	n	%	n	%	n	%	
Sex	Female	41	22.91	138	77.09	0.348*	66	36.87	8	4.47	0	0.00	2	1.12	8	4.47	15	8.38	5	2.79	42	23.46	2	1.12	8	4.47	19	10.61	4	2.23	0.069*
	Male	39	27.46	103	72.54		48	33.80	7	4.93	0	0.00	7	4.93	8	5.63	6	4.23	0	0.00	26	18.31	4	2.82	14	9.86	16	11.27	6	4.23	
Age group	01 to 10 years	1	14.29	6	85.71	0.527*	3	42.86	0	0.00	0	0.00	0	0.00	0	0.00	1	14.29	0	0.00	2	28.57	0	0.00	0	0.00	1	14.29	0	0.00	0.477*
	11 to 20 years	20	24.69	61	75.31		26	32.10	2	2.47	0	0.00	5	6.17	6	7.41	5	6.17	1	1.23	13	16.05	1	1.23	3	3.70	15	18.52	4	4.94	
	21 to 30 years	13	23.64	42	76.36		18	32.73	2	3.64	0	0.00	4	7.27	3	5.45	1	1.82	0	0.00	9	16.36	1	1.82	4	7.27	11	20.00	2	3.64	
	31 to 40 years	12	22.64	41	77.36		18	33.96	1	1.89	0	0.00	0	0.00	3	5.66	2	3.77	2	3.77	16	30.19	2	3.77	6	11.32	2	3.77	1	1.89	
	41 to 50 years	14	32.56	29	67.44		18	41.86	2	4.65	0	0.00	0	0.00	2	4.65	3	6.98	1	2.33	7	16.28	1	2.33	4	9.30	4	9.30	1	2.33	
	51 to 60 years	5	16.13	26	83.87		10	32.26	3	9.68	0	0.00	0	0.00	1	3.23	1	3.23	1	3.23	9	29.03	1	3.23	3	9.68	1	3.23	1	3.23	
	61 to 70 years	5	20.00	20	80.00		10	40.00	2	8.00	0	0.00	0	0.00	1	4.00	4	16.00	0	0.00	6	24.00	0	0.00	1	4.00	0	0.00	1	4.00	
	≥ to 71 years	10	38.46	16	61.54		11	42.31	3	11.54	0	0.00	0	0.00	0	0.00	4	15.38	0	0.00	6	23.08	0	0.00	1	3.85	1	3.85	0	0.00	
Total		80	24.92	241	75.08		114	35.51	15	4.67	0	0.00	9	2.80	16	4.98	21	6.54	5	1.56	68	21.18	6	1.87	22	6.85	35	10.91	10	3.13	

n: absolute frequency. %: relative frequency.

*Chi-square test. **Chi-square test corrected by yates

This study analyzes variables such as sex and age group in relation to the recurrence, recurrence time, and anatomical site of keratocysts. Regarding recurrence by sex, it was more frequent in men, with 20 cases (14.08%), and less frequent in women, with 17 cases (9.50%). The p-value was 0.201, indicating that this difference was not statistically significant. With respect to recurrence distribution by age group, the highest percentage of recurrences occurred in the 11-20-year group, with 10 cases (12.35%), while no recurrences were reported in individuals aged ≥71 years, showing a statistically significant difference (p < 0.001). When analyzing recurrence time by sex, the highest number of recurrence cases occurred within the first year, being more frequent in men with 11 cases (7.75%) compared to women with 6 cases (3.35%). Recurrences occurring ≥5 years were also more common in men, with 5 cases (3.52%), compared to 2 cases in women (1.12%). The difference between sexes was not statistically significant (p = 0.219). Regarding the distribution of age group relative to recurrence time, the age groups with the highest number of cases were 11-20 years and 21-30 years, with 6 cases each. Statistical analysis revealed that variations in recurrence time among age groups were not significant (p = 0.075). When comparing specific recurrence site by sex, both women and men showed the highest number of recurrences in the mandibular body region, with 7 cases each. The p-value was 0.727, indicating that the difference was not statistically significant. When analyzing specific recurrence site by age group, the highest number of cases was observed in the mandibular body, with 4 cases in the 21-30-year group. The difference between specific recurrence site and age group was not statistically significant (p = 0.34) (Table 3)

**Table 3:** Recurrence of keratocysts in the maxillofacial pathology laboratory of the UPCH teaching dental center, Lima-Peru during the period from 2000 to 2019

Variables		RECURRENCE				RECURRENCE TIME					PLACE OF RECURRENCE					
		No		Yes		P	Not present		Less than a year		1 year		2 years		3 years		4 years		≥ 5 years		p	No specific location		Incisors and canines		Premolars		Molars (upper)		Other areas of the maxilla		Symphysis		Parasymphysis		Body		Angle		Branch		Molars (lower)		Other areas of the mandible		p	
		n	%	n	%		n	%	n	%	n	%	n	%	n	%	n	%	n	%		n	%	n	%	n	%	n	%	n	%	n	%	n	%	n	%	n	%	n	%	n	%	n	%	
Sex	Female	162	90.50	17	9.50	0.201*	162	90.50	0	0.00	6	3.35	3	1.68	4	2.23	2	1.12	2	1.12	0.219*	162	90.50	1	0.56	1	0.56	0	0.00	1	0.56	1	0.56	0	0.00	7	3.91	0	0.00	2	1.12	4	2.23	0	0.00	0.727*
	Male	122	85.92	20	14.08		122	85.92	0	0.00	11	7.75	1	0.70	1	0.70	2	1.41	5	3.52		122	85.92	1	0.70	2	1.41	2	1.41	0	0.00	1	0.70	0	0.00	7	4.93	1	0.70	3	2.11	3	2.11	0	0.00	
Age group	01 to 10 years	2	28.57	5	71.43	<0.001**	2	28.57	0	0.00	2	28.57	0	0.00	1	14.29	1	14.29	1	14.29	0.075**	2	28.57	0	0.00	0	0.00	0	0.00	0	0.00	1	14.29	0	0.00	2	28.57	0	0.00	0	0.00	2	28.57	0	0.00	0.344**
	11 to 20 years	71	87.65	10	12.35		71	87.65	0	0.00	6	7.41	1	1.23	1	1.23	0	0.00	2	2.47		71	87.65	1	1.23	1	1.23	2	2.47	0	0.00	0	0.00	0	0.00	1	1.23	0	0.00	2	2.47	3	3.70	0	0.00	
	21 to 30 years	46	83.64	9	16.36		46	83.64	0	0.00	6	10.91	2	3.64	0	0.00	1	1.82	0	0.00		46	83.64	0	0.00	1	1.82	0	0.00	1	1.82	0	0.00	0	0.00	4	7.27	1	1.82	2	3.64	0	0.00	0	0.00	
	31 to 40 years	48	90.57	5	9.43		48	90.57	0	0.00	1	1.89	0	0.00	1	1.89	1	1.89	2	3.77		48	90.57	1	1.89	0	0.00	0	0.00	0	0.00	1	1.89	0	0.00	3	5.66	0	0.00	0	0.00	0	0.00	0	0.00	
	41 to 50 years	39	90.70	4	9.30		39	90.70	0	0.00	1	2.33	0	0.00	1	2.33	1	2.33	1	2.33		39	90.70	0	0.00	0	0.00	0	0.00	0	0.00	0	0.00	0	0.00	2	4.65	0	0.00	1	2.33	1	2.33	0	0.00	
	51 to 60 years	28	90.32	3	9.68		28	90.32	0	0.00	1	3.23	0	0.00	1	3.23	0	0.00	1	3.23		28	90.32	0	0.00	0	0.00	0	0.00	0	0.00	0	0.00	0	0.00	2	6.45	0	0.00	0	0.00	1	3.23	0	0.00	
	61 to 70 years	24	96.00	1	4.00		24	96.00	0	0.00	0	0.00	1	4.00	0	0.00	0	0.00	0	0.00		24	96.00	0	0.00	1	4.00	0	0.00	0	0.00	0	0.00	0	0.00	0	0.00	0	0.00	0	0.00	0	0.00	0	0.00	
	≥ to 71 years	26	100	0	0.00		26	100	0	0.00	0	0.00	0	0.00	0	0.00	0	0.00	0	0.00		26	####	0	0.00	0	0.00	0	0.00	0	0.00	0	0.00	0	0.00	0	0.00	0	0.00	0	0.00	0	0.00	0	0.00	
Total		284	88.47	37	11.53		88.5	88.47	0	0.00	17	5.30	4	1.25	5	1.55	4	1.25	7	2.18		284	88.47	2	0.62	3	0.93	2	0.62	1	0.31	2	0.62	0	0.00	14	4.37	1	0.31	5	1.57	7	2.18	0	0.00	

n: absolute frequency. %: relative frequency.

*Chi-square test. **Chi-square test corrected by Yates

## DISCUSSION

According to the fifth edition of the WHO Classification of Head and Neck Tumors (2022), the odontogenic keratocyst (OKC) is currently defined as an epithelial cystic lesion derived from the enamel organ or remnants of the dental lamina ^3^. In the present study, which analyzed 321 cases diagnosed between 2000 and 2019 at the Oral and Maxillofacial Pathology Laboratory of Universidad Peruana Cayetano Heredia (UPCH), relevant clinical, epidemiological, and therapeutic patterns were identified.

The findings confirm a marked predilection for the mandible (75.08%), particularly the mandibular body and angle, which is consistent with reports describing mandibular involvement ranging from 70% to 80% ^18^^,^^19^. This distribution may be explained by the persistence of epithelial remnants related to odontogenesis in the posterior mandibular region ^19^.

Regarding recurrence, 11.53% of the cases in the present series exhibited lesion reappearance. This finding agrees with previous studies reporting recurrence rates ranging widely from 0% to 62% ^20^^,^^23^. Stoelinga reported that most recurrences occur within the first five years after treatment, highlighting the importance of long-term follow-up ^20^. Other authors have emphasized that the infiltrative biological behavior and high proliferative potential of OKCs contribute significantly to incomplete eradication and subsequent recurrence ^15^^,^^21^.

Treatment of odontogenic keratocysts remains controversial due to their variable clinical behavior and recurrence risk. Several therapeutic approaches have been described, including enucleation, marsupialization, curettage, peripheral ostectomy, application of chemical agents, and segmental resection, with treatment choice depending on lesion size, location, and relationship with adjacent anatomical structures ^9^^,^^10^^,^^26^. Adequate preoperative planning and individualized case assessment are essential to optimize outcomes and minimize complications.

Marsupialization and decompression share the common goal of reducing cystic volume and intracystic pressure but differ in their surgical approach ^14^. Marsupialization involves partial removal of the cyst wall and suturing the lesion margins to the oral mucosa, creating a permanent communication with the oral cavity ^14^. Decompression, in contrast, consists of maintaining a drainage opening that allows continuous fluid evacuation and pressure reduction, promoting bone regeneration ^12^^,^^13^. Both techniques induce favorable biological changes that facilitate subsequent enucleation and reduce surgical morbidity ^12^^−^^14^.

More aggressive approaches, such as resection, have been proposed as therapeutic options; however, their routine use remains controversial due to high morbidity and postoperative complications, despite reports of very low recurrence rates ^10^^,^^23^. In recent years, the use of topical 5-fluorouracil (5-FU) has been proposed as an alternative adjuvant therapy. Akhter Lone et al. reported that 5-FU may offer advantages over Carnoy’s solution, including technical simplicity, reduced operative time, lower morbidity, cost-effectiveness, and minimal recurrence; nevertheless, further large-scale clinical trials are required to validate these findings ^27^.

The absence of universally accepted treatment protocols has prompted ongoing investigation into less invasive strategies with comparable success rates. A systematic review by Gonçalves et al. reported that enucleation remains the most frequently used technique but is associated with recurrence rates of 30% to 60% when not combined with adjuvant methods, whereas marsupialization showed lower recurrence rates ^24^. Karaca et al. demonstrated that combining enucleation with peripheral ostectomy significantly reduced recurrence rates ^25^.

Simple enucleation alone is generally not recommended due to high recurrence rates ^16^. The use of adjuvant techniques, such as Carnoy’s solution or cryotherapy, has been shown to significantly reduce recurrence ^9^^,^^10^. Recurrence may result from incomplete enucleation or curettage due to the fragile cyst wall, persistence of epithelial remnants, satellite cysts, or invasion into adjacent soft tissues ^15^^,^^21^.

Several studies have identified risk factors associated with recurrence, including the surgical technique employed, incomplete curettage, and the presence of epithelial islands or microcysts ^15^^,^^21^^,^^26^. Additionally, the infiltrative capacity of OKCs and their tendency to extend into surrounding soft tissues further complicate complete surgical removal ^21^. Histopathological features such as satellite cysts and multilocular radiographic patterns have also been associated with increased recurrence risk ^15^.

In the present study, a slight predominance in female patients was observed. Although some studies report male predominance, descriptive patterns suggest that adolescents and young adults are the most commonly affected group ^4^^,^^5^. This population may particularly benefit from conservative surgical strategies and prolonged follow-up.

Most recurrences in this study occurred within the first year after treatment, corroborating previous reports emphasizing the critical importance of rigorous early postoperative monitoring ^20^^,^^23^. Recurrences were predominantly located in the mandibular body and molar region, areas that present greater surgical complexity due to proximity to the mandibular canal and dense bone architecture, factors that may favor persistence of epithelial remnants ^19^^,^^21^.

Although many variables did not reach statistical significance, the observed descriptive patterns underscore the need for larger, prospective studies with standardized follow-up protocols. Future research should focus on evaluating the influence of histological subtypes, surgical techniques, and molecular factors on OKC behavior and recurrence ^3^^,^^15^^,^^21^.

## CONCLUSION

In conclusion, odontogenic keratocysts diagnosed at the Oral and Maxillofacial Pathology Laboratory of the Teaching Dental Center of the Universidad Peruana Cayetano Heredia during the 2000-2019 period represent a relatively uncommon entity among the total lesions analyzed. Their low prevalence confirms that, although biologically significant due to their locally aggressive behavior and potential for recurrence, they do not constitute one of the most frequent cystic pathologies encountered in routine oral pathology practice within this Peruvian population.

The epidemiological pattern observed suggests a predominance in younger patients and a marked mandibular involvement, reinforcing the importance of thorough clinical and radiographic assessment, particularly in early stages of life. Although no statistically significant differences were identified in most analyses according to sex, age group, or anatomical location, the concentration of cases in specific age ranges and anatomical regions may indicate the influence of developmental and biological factors in their pathogenesis. These trends, while not statistically conclusive, remain clinically relevant.

Regarding recurrence, the findings indicate that, although the overall recurrence rate was not high, it remains clinically significant and occurred predominantly within the first year following treatment. This emphasizes the need for strict and structured follow-up protocols, especially in younger patients, to ensure early detection of recurrences and to optimize long-term prognosis. Collectively, these results provide valuable local epidemiological evidence that may contribute to improving diagnostic strategies, therapeutic planning, and follow-up protocols within the Peruvian clinical context, while also enriching the limited regional literature on odontogenic keratocysts.
